# A public health risk model using prior healthcare exposures identifies healthcare-associated pathogen carriage

**DOI:** 10.1017/ice.2026.10397

**Published:** 2026-03

**Authors:** Sarah E. Sansom, Tanner Shull, Mary K. Hayden, Michael Schoeny, Angela Tang, Mai Vue, Anh-Thu Runez, Dejan Jovanov, William E. Trick, Michael Y. Lin

**Affiliations:** 1 Department of Internal Medicine, Rush University, Chicago, IL, USA; 2 College of Nursing, Rush University, Chicago, IL, USA; 3 Illinois Department of Public Health, Chicago, IL, USA; 4 Department of Medicine, Cook County Health, Chicago, IL, USA

## Abstract

**Background:**

Early identification of patients colonized with multidrug-resistant organisms (MDROs) facilitates infection control interventions. We assessed a Public Health Risk Model’s ability to predict carbapenem-resistant Enterobacterales and other MDROs.

**Methods:**

We retrospectively analyzed a medical intensive care unit patient cohort screened at time of admission for MDRO carriage (1/2017–1/2018). Encounters were linked to Illinois Hospital Discharge Data and assigned a public health risk model probability score. We compared the model’s performance to traditional screening strategies that use variables locally available to clinicians at time of admission (i.e., transfer from other hospital, tracheostomy, gastrostomy, pressure ulcer). Model discrimination was evaluated by quantifying the area under the curve (AUC). For each approach, we assessed sensitivity, specificity, and number needed to screen (NNS) to detect one MDRO carrier.

**Results:**

Model probability calculation was successful in 1237/1250 (98.9%) admissions. The model identified carbapenem-resistant Enterobacterales colonization well (AUC 0.82) and generalized to predict colonization with other healthcare-associated MDROs, including carbapenem-resistant *Pseudomonas aeruginosa* (AUC 0.82) and vancomycin-resistant enterococci (AUC 0.76). The model did not predict MDROs with known local community reservoirs, i.e., third-generation cephalosporin-resistant Enterobacterales (AUC 0.61) and methicillin-resistant *Staphylococcus aureus* (AUC 0.59). At the same NNS, the model had higher sensitivity compared to use of traditional screening strategies (68% versus 41%).

**Conclusion:**

A risk model using patient-level healthcare exposure data from a state public health dataset identified critically ill patients likely to harbor healthcare-associated MDROs at the time of admission.

## Introduction

Screening newly admitted patients for multidrug-resistant organism (MDRO) colonization may be an important component of prevention strategies and inform empiric antimicrobial selection, but is resource-intensive.^1,2^ Given the low overall prevalence of some MDROs (e.g., carbapenem-resistant Enterobacterales, carbapenem-resistant *Pseudomonas aeruginosa*) in many healthcare settings,^3^ a targeted screening approach that stratifies risk for MDRO colonization at the time of admission may be more practical and cost-effective for screening programs.^4^

In the U.S., prior healthcare exposure is a major risk factor for colonization with MDROs and could potentially be leveraged to risk-stratify patients for targeted MDRO screening.^5^ At the institutional level, admission assessment of prior healthcare exposure is difficult due to missing information within the medical record or limited communication between healthcare facilities at the time of patient transfer.^4,6^ In contrast, a centralized public health model has the advantage of being able to efficiently capture patients’ prior exposure to all healthcare facilities in the jurisdiction, with the trade-off that capture of clinical variables may be limited to billing codes (i.e., ICD codes).

We previously developed and validated a Public Health Risk Model to identify carbapenem-resistant Enterobacterales colonization at the time of hospital admission using patient-level healthcare exposure data in the Illinois Hospital Discharge Dataset.^7^ In the original derivation of the model, we identified age, acute care hospitalizations in the prior 365 days (both short-term and long-term hospitals), and ICD-coded infection diagnoses as independent risk factors for carbapenem-resistant Enterobacterales colonization at the time of hospital admission.^7^

In this study, we performed a comprehensive validation of the Public Health Risk Model in a medical intensive care unit cohort. Our study cohort was screened for multiple MDROs at the time of medical intensive care unit admission, resulting in robust characterization of MDRO colonization status.^3^ In addition to validating model performance to predict carbapenem-resistant Enterobacterales colonization, we also tested the hypothesis that the model would be generalizable to predict colonization by other MDROs due to shared risk factors.^8^ Additionally, we compared the performance of the model to traditional screening strategies that use locally captured clinical variables to target screening.

## Methods

### Setting, participants, and study covariates

We conducted a retrospective model validation study at Rush University Medical Center, a tertiary-care medical center in Chicago, Illinois. The study cohort comprised patients ≥18 years old who were admitted to the 25-bed medical intensive care unit from January 11, 2017 through January 10, 2018 and who had been prospectively cultured for MDROs via rectal or fecal swab within 48 hours of admission.^3^ Our region is endemic for the MDROs of interest; MDROs of interest included carbapenem-resistant Enterobacterales, carbapenem-resistant *Pseudomonas aeruginosa*, carbapenem-resistant *Acinetobacter baumannii*, vancomycin-resistant enterococci, and third-generation cephalosporin-resistant Enterobacterales. Methicillin-resistant *Staphylococcus aureus* nasal colonization at admission was determined by routine clinical screening, as required per Illinois law under the MRSA Screening and Reporting Act [210 ILCS 83]. Carbapenem-resistant Enterobacterales, carbapenem-resistant *P. aeruginosa,* and carbapenem-resistant *A. baumannii* were further classified as a composite group of carbapenem-resistant organisms (CROs) in our analyses. CROs were defined as (1) Enterobacterales isolates exhibiting phenotypic resistance to ertapenem, imipenem or meropenem, or (2) *P. aeruginosa* or *A. baumannii* isolates exhibiting phenotypic resistance to imipenem or meropenem. Minimal inhibitory concentration results were interpreted using Clinical Laboratory Standards Institute breakpoints.^9^

### Data linkage and public health model risk score generation

Each patient’s index medical intensive care unit admission from the validation cohort was linked to the Illinois Hospital Discharge Dataset,^10^ with secure data transfer between institutions using the open-source privacy-preserving record linkage system Linkja.^11^ The Illinois Hospital Discharge Dataset contains comprehensive encounter-level information for all Illinois hospitalizations in short-term and long-term acute care hospitals. Hospital encounter data in this dataset included patient identifiers, facility identifiers, and encounter characteristics (e.g., dates of hospital admission and discharge, diagnosis codes, and procedure codes). By applying the coefficients from our prior Public Health Risk Model,^7^ the Illinois Department of Public Health retrospectively calculated the probability of MDRO colonization at the time of medical intensive care unit admission for individuals in our cohort. Covariates included age and, during the prior 365 days, number of short-term acute care hospitalizations and mean length of stay, number of long-term acute care hospitalizations and mean length of stay, and prior hospital admission with an ICD-10 diagnosis code indicating probable bacterial infection.^7^ Calculation of the Public Health Risk Score was centralized within state public health bioinformatics infrastructure and was reported as a percent. The linked dataset was de-identified and model inputs were opaque to the research team.

### Statistical analyses

Demographic and clinical characteristics were expressed as frequencies with proportions and medians with interquartile ranges, as appropriate. Public Health Risk Model performance to predict MDROs was evaluated by applying the frozen coefficients from the prior model using logistic regression. Model discrimination for MDRO colonization was evaluated as area under the curve (AUC) using receiver operating characteristic (ROC) curves. Model performance was further evaluated by calibration plot and Brier score.

We compared performance of the model for identification of MDRO colonization to traditional screening strategies utilizing patient clinical characteristics typically available to clinicians at time of admission: (1) Hospital transfer (from a short-term or long-term acute care hospital), (2) Presence of either a tracheostomy or a percutaneous gastronomy tube, and (3) Presence of a pressure ulcer.^12,13^ The presence of tracheostomy, gastrostomy tube, or pressure ulcer was assessed through daily prospective review of bedside medical records at the time of initial cohort enrollment. Transfer from other hospital was determined retrospectively from the electronic medical record.

Missing values for the model probability (*n* = 13) after data linkage were assigned a value of 0, as that would be consistent with the value assigned when operationalized. Association of traditional screening strategies with MDRO colonization were evaluated by *χ* ^2^ testing. Sensitivity was calculated as the proportion of true positives identified using each approach compared to the reference standard of all patients evaluated by culture detection at the time of medical intensive care unit admission. Specificity was calculated as the proportion of true negatives by each approach compared to the reference standard. The Number Needed to Screen (NNS) to detect one carrier was calculated as the inverse of the positive predictive value. For all calculations, statistical significance was defined as a two-tailed *P*-value < .05. Statistical analysis was performed using SAS version 9.4 (Cary, NC). Default statistical tests in SAS were used for generation of confidence intervals, including the Hanley and McNeil method for AUC confidence intervals and the asymptotic Wald method for sensitivity.

## Results

### The public health risk model predicted carbapenem-resistant enterobacterales and healthcare-associated MDRO colonization

The study cohort included 1,250 unique participants, of whom 1,237 (98.9%) were successfully linked to the Illinois Hospital Discharge Dataset. Using the model, most admission-level risk scores were low (median 0.3%, IQR 0.2–0.4%, range 0–74.6%); only 19.2% (*n* = 238) had a measured risk of ≥ 0.5%, which we determined post hoc as the threshold that optimized the balance between sensitivity and specificity. Among these 1,237 admissions, 27 originally tested positive for carbapenem-resistant Enterobacterales carriage, of which 17 had detection of a carbapenemase gene (16 *blaKPC* and 1 *blaNDM*). Regarding other MDROs, 10 individuals tested positive for carbapenem-resistant *P. aeruginosa* (of which all were negative for presence of carbapenemase genes), 160 positive for vancomycin-resistant enterococci, 217 positive for third-generation cephalosporin-resistant Enterobacterales, 53 positive for methicillin-resistant *S. aureus*, and 1 positive for carbapenem-resistant *A. baumannii*. The patient colonized with *A. baumannii* was co-colonized with carbapenem-resistant Enterobacterales.

The model discriminated well for colonization with carbapenem-resistant Enterobacterales at the time of admission (AUC 0.82, 95% confidence interval [CI] 0.72–0.91). The model also discriminated well for colonization with other healthcare-associated MDROs (i.e., carbapenem-resistant *P. aeruginosa*, vancomycin-resistant enterococci). The model performed poorly to predict MDROs with known local community reservoirs (i.e., third-generation cephalosporin-resistant Enterobacterales, methicillin-resistant *S. aureus*) (Table 1, Figure S1).

**Table 1.** Application of the public health risk model to identify MDROs

Multidrug-resistant organism of interest	Encounters with MDRO detected at admission *n*, % (N = 1,237)	Area under the receiver operating characteristic curve (95% CI)
**Healthcare-associated pathogens**		
Carbapenem-resistant Enterobacterales	27 (2.2)	0.82 (0.72–0.91)
Carbapenem-resistant *Pseudomonas aeruginosa*	10 (0.8)	0.82 (0.66–0.97)
Any carbapenem-resistant organism	37 (3.0)	0.81 (0.74–0.90)
Vancomycin-resistant Enterococcus	160 (12.9)	0.76 (0.72–0.80)
**Pathogens with local community reservoirs**		
3^rd^-generation cephalosporin-resistant Enterobacterales	217 (17.5)	0.61 (0.57–0.65)
Methicillin-resistant *Staphylococcus aureus*	53 (4.3)	0.59 (0.51–0.67)

### The public health risk model performed better than traditional screening strategies for CRO colonization

**Figure 1.**
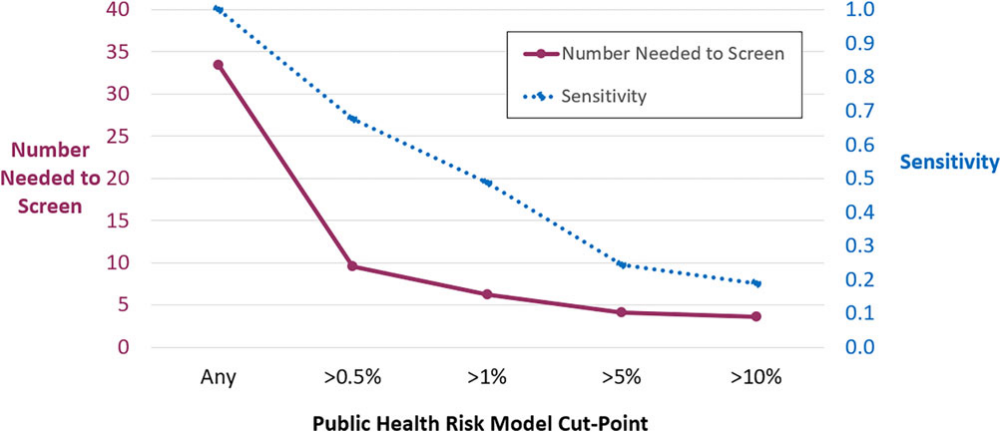
Determination of public health risk model thresholds to predict carbapenem-resistant organism (CRO) colonization. Various thresholds to apply the model to identify patients with CRO colonization were evaluated using number needed to screen (left Y-axis; purple solid line) and sensitivity (right Y-axis; blue dotted line). The absolute risk inferred from the number needed to screen appears higher than the predicted modeled risk because the risk model was originally calibrated to a lower CRO prevalence population (all hospitalized patients in Illinois), whereas the current validation utilized a higher prevalence cohort (intensive care unit patients).

We next assessed performance of the model compared to traditional screening strategies. We first explored various thresholds to apply the model to identify patients with CRO colonization. Higher risk score cutoff was inversely related to screening sensitivity (Figure 1). A sensitivity table exploring different cutoff values is also shown in Supplemental Table 1. We identified a threshold of ≥ 0.5% as reasonably balanced between NNS and sensitivity for a screening test. Metrics to assess discrimination and calibration were generally favorable (intercept –3.60, slope 5.76, c-statistic/AUC 0.82, Likelihood Ratio *P* = .001, Wald *P* < .001, Brier score 0.029; model diagnostics shown in Supplemental Figure 2).

**Figure 2.**
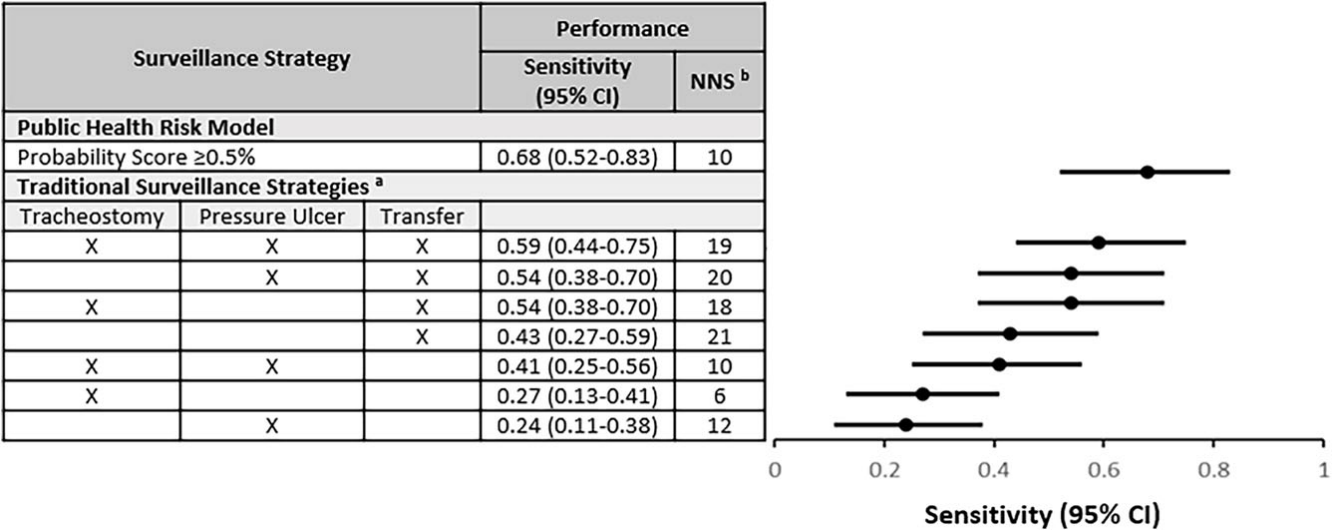
Sensitivity of targeted carbapenem-resistant organism (CRO) screening strategies. Sensitivity and number needed to screen of different strategies were compared. The sensitivities of traditional screening strategies were lower compared to the public health risk model for identification of CRO colonization. ^a^For clinical risk factors, the presence of any one risk factor would trigger screening. ^b^Number needed to screen to detect one CRO carrier was calculated as the inverse of the positive predictive value.

Next, we evaluated the traditional screening strategies that utilize patient risk factors at time of admission, including hospital transfer, presence of a tracheostomy or percutaneous gastrostomy tube, and presence of a pressure ulcer. We found that hospital transfer (*P* = .02), tracheostomy (*P* < .001), and presence of pressure ulcer (*P* < .001) were each associated with CRO colonization (Table 2). We then compared the sensitivity and NNS to detect one CRO carrier using traditional screening strategies to the Public Health Risk Model. The sensitivities of traditional screening strategies were lower than that of the model for identification of CRO colonization (Figure 2). Diagnostic measures for each strategy are shown in Supplemental Table 2.

**Table 2.** Traditional patient risk factors for CRO colonization

Risk factor at time of admission	Colonized with CRO N = 37	Not colonized with CRO N = 1,213	Odds ratio (95% confidence interval)	*P*-Value
Hospital transfer (*n* = 332)	16 (43)	316 (26)	2.16 (1.11–4.20)	***.02***
Presence of tracheostomy (*n* = 56)	10 (27)	46 (4)	9.29 (4.25–20.33)	***< .01***
Presence of gastrostomy tube (*n* = 14)	0 (0)	14 (1)	*n/a*	*.51*
Presence of pressure ulcer (*n* = 163)	14 (38)	149 (12)	4.35 (2.19–8.63)	***< .01***

## Discussion

We found that a risk-based prediction model using patient-level healthcare exposure data from a state public health dataset^7^ could discriminate critically ill patients more likely to harbor healthcare-associated MDROs at the time of hospital admission. We confirmed that the Public Health Risk Model could predict a patient’s carbapenem-resistant Enterobacterales colonization status at time of admission, and for the first time, we demonstrated the model’s generalizability to other healthcare-associated MDROs, including carbapenem-resistant *P. aeruginosa* and vancomycin-resistant enterococci. The model did not predict MDROs with significant local community reservoirs of transmission, including third-generation cephalosporin-resistant Enterobacterales and methicillin-resistant *S. aureus*. The sensitivity of the model for discrimination of CRO carriage exceeded that of traditional screening strategies.

The use of a centralized state public health database for MDRO prediction can overcome major limitations in data sharing that occur when patients receive care across multiple healthcare institutions. For example, prior long-term acute care hospital exposure has been identified as one of the strongest risk factors for colonization with MDROs.^14,15^ However, for patients transferred directly from a long-term acute care hospital to our hospital, we have not identified a local electronic method to reliably distinguish long-term acute care hospital from other hospitals as the sending facility.^4^ Furthermore, patients with long-term acute care hospital exposure in the prior year may have had intervening care at a nursing home or have returned home prior to readmission to our hospital, rendering point-of-origin billing codes or clinical documentation at time of admission less accurate. In a multicenter retrospective study, Goodman et al. found similar limitations in identifying prior long-term care facility exposure using only electronic billing codes or clinical documentation at their institution, highlighting the value of a statewide database to identify prior healthcare exposures.^6^ Although there was success with the Illinois Department of Public Health, challenges to use of a centralized state public health database include state-to-state variability in the accessibility of such a dataset and the internal capacity to deploy the prediction model’s parameters by personnel at public health departments.

Targeted screening strategies should be tailored to each institution based on available resources and local MDRO epidemiology.^16^ We demonstrated that traditional screening approaches are useful to evaluate risk of CRO colonization and may be used in healthcare settings where state public health databases are not readily available. Some clinical variables, such as presence of a tracheostomy or pressure ulcer, are easily obtained through physical examination. Our findings align with prior studies demonstrating variable success in identifying patients with CROs using clinical risk factors. Frequently identified risk factors include prior healthcare exposure (e.g., hospitalization, long-term care residency),^4,7,14,15,17–19^ exposure to antimicrobial agents,^7,15,18–20^ invasive devices or procedures (e.g., mechanical ventilation, tracheostomy, central line),^15,18,21^ presence of pressure ulcers,^22^ and history of MDROs .^15,20,21^ The variation in identified CRO risk factors across models may be attributable to host and organism heterogeneity, differences in regional CRO epidemiology, CRO misclassification, and limitations of data capture by the electronic health record.^15^

Our study demonstrates the potential utility of a public health database to improve infection prevention. Public health departments may provide actionable information to local healthcare institutions and providers that extends beyond traditional sharing of reportable diseases. In Illinois, we are testing near-real time automated notification of high-risk patients identified through the current Public Health Risk Model to admitting healthcare facilities using an existing alerting infrastructure (the Illinois XDRO registry^23^). The statewide discharge database used to generate these risk scores is updated quarterly, which may introduce a lag of up to 3 months in available data for risk score generation. Although several of the strategies evaluated in our study had limited sensitivity to identify CRO carriage, this does not negate their usefulness in selecting populations for targeted screening. For example, a facility with very limited resources could prioritize a higher specificity approach by performing CRO screening for patients admitted with tracheostomies, which required screening of only 6 individuals to identify one CRO carrier in our cohort.

There are several limitations that should be considered when interpreting the results of our study. First, we considered culture detection of MDRO colonization to be the reference standard for this study, though culture detection may be less sensitive than molecular detection methods when organism burden is low.^3,24^ Second, our study cohort comprised medical intensive care unit patients in a region where CROs are endemic.^4,25,26^ Thus, our findings may not be generalizable to non-critically ill populations or to other geographic regions. Third, the Illinois Hospital Discharge Dataset has current limitations that influence its ability to accurately inform risk prediction, namely that data updates occur quarterly with lags up to 6 months, and lack of encounter data from skilled nursing facilities.

In conclusion, we found that a risk model based on a state-level public health hospital discharge database could assess risk in critically ill patients for colonization with healthcare-associated MDROs, demonstrating improved sensitivity over traditional screening strategies.

## Supporting information

Sansom et al. supplementary material Sansom et al. supplementary material

## Supplementary material

The supplementary material for this article can be found at https://doi.org/10.1017/ice.2026.10397
