# Supplementary material for: A public health risk model using prior healthcare exposures identifies healthcare-associated pathogen carriage
Source: Infect Control Hosp Epidemiol. 2026 Jan 21;47(3):272–6. doi: 10.1017/ice.2026.10397 (PMC12885053; doi:10.1017/ice.2026.10397)

SUPPLEMENTAL MATERIALS

Supplemental Table 1. Sensitivity Table of Model Cut-Points

Cut-point (%)	Sensitivity	Specificity	True Positive	True Negative	False Positive	False Negative	Positive Predictive Value	Number Needed to Screen
**10.00**	0.189	0.985	7	1182	18	30	0.28	3.57
**5.00**	0.243	0.976	9	1171	29	28	0.24	4.22
**4.00**	0.270	0.975	10	1170	30	27	0.25	4.00
**3.00**	0.270	0.965	10	1158	42	27	0.19	5.20
**2.00**	0.459	0.948	17	1138	62	20	0.22	4.65
**1.00**	0.486	0.922	18	1106	94	19	0.16	6.22
**0.90**	0.486	0.918	18	1101	99	19	0.15	6.50
**0.80**	0.486	0.908	18	1089	111	19	0.14	7.17
**0.70**	0.541	0.895	20	1074	126	17	0.14	7.30
**0.60**	0.595	0.880	22	1056	144	15	0.13	7.55
**0.50**[a]	0.676	0.823	25	987	213	12	0.11	9.52
**0.44**[b]	0.784	0.776	29	931	269	8	0.10	10.28
**0.40**	0.784	0.728	29	874	326	8	0.08	12.24
**0.30**	0.865	0.539	32	647	553	5	0.05	18.28
**0.20**	0.919	0.343	34	412	788	3	0.04	24.18
**0.10**	0.973	0.161	36	193	1007	1	0.03	28.97

[a] Selected cut-point for downstream analyses, highlighted in yellow
[b] Calculated Youden cut-point, highlighted in green

Supplemental Table 2. Diagnostic Accuracy of Targeted CRO Screening Strategies

Strategy [a]	True positive	Total positive	PPV	True negative	Total negative	NPV	Sensitivity (95% CI)	Specificity (95% CI)
Public Health Risk Model ≥0.5%	25	238	0.11	987	1012	0.98	0.68 (0.52-0.83)	0.81 (0.69-0.94)
Tracheostomy + Pressure ulcer + Hospital Transfer	22	421	0.05	801	829	0.97	0.59 (0.44-0.75)	0.66 (0.51-0.81)
Tracheostomy + Hospital transfer	20	363	0.06	857	887	0.97	0.54 (0.38-0.70)	0.71 (0.56-0.85)
Pressure ulcer + Hospital transfer	20	401	0.05	832	849	0.98	0.54 (0.38-0.70)	0.69 (0.54-0.84)
Hospital transfer	16	333	0.05	896	917	0.98	0.43 (0.27-0.59)	0.74 (0.60-0.88)
Tracheostomy + Pressure ulcer	15	155	0.10	1060	1095	0.97	0.41 (0.25-0.56)	0.87 (0.77-0.98)
Tracheostomy	10	56	0.18	1154	1194	0.97	0.27 (0.13-0.41)	0.95 (0.88-1.00)
Pressure Ulcer	9	110	0.08	1103	1140	0.97	0.24 (0.11-0.38)	0.91 (0.82-1.00)

[a] *For clinical risk factors, the presence of any one risk factor would trigger screening*

Supplemental Figure 1. Application of the Public Health Risk Model to Identify Healthcare-Associated Multidrug-Resistant Organisms (MDRO). The model performed well to identify critically-ill patients with healthcare-associated MDROs. Receiver operator curves (ROC) are shown with number of positive screening cases by culture (n), area under the curve (AUC) and 95% confidence intervals. The outcome of carbapenem-resistant organisms (CRO) included a composite of carbapenem-resistant Enterobacterales, carbapenem-resistant *Pseudomonas aeruginosa*, and carbapenem-resistant *Acinetobacter baumannii*.

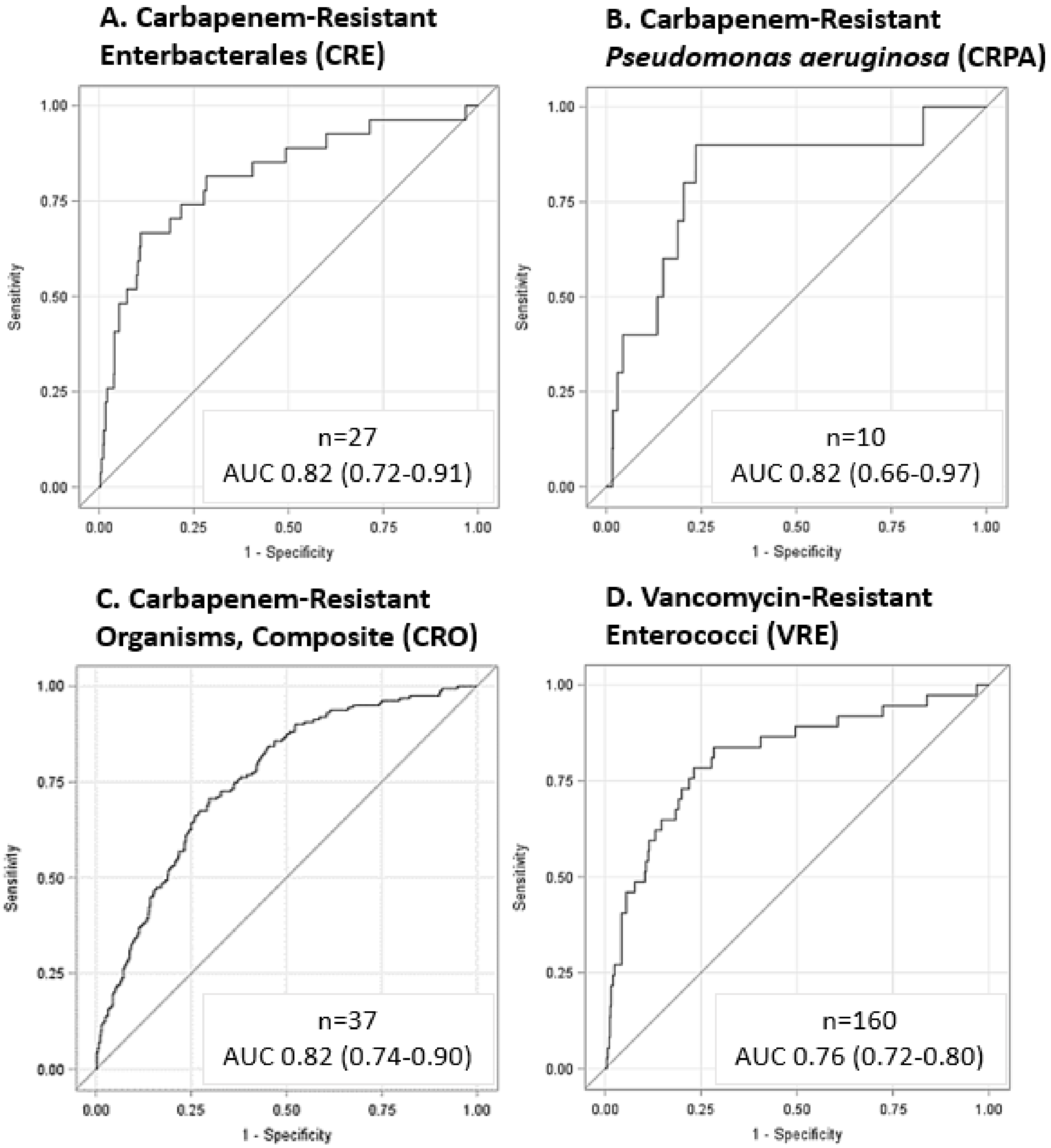

Supplemental Figure 2. Model Diagnostics. Metrics to assess discrimination and calibration were generally favorable. The model showed strong predictive significance (intercept -3.60, slope 5.76, c-statistic/AUC 0.82, Likelihood Ratio p=0.001, Wald p<0.001) to discriminate cases. The Brier score was low (0.029), confirming good performance.

A. Calibration Plot: Observed vs Predicted Probability

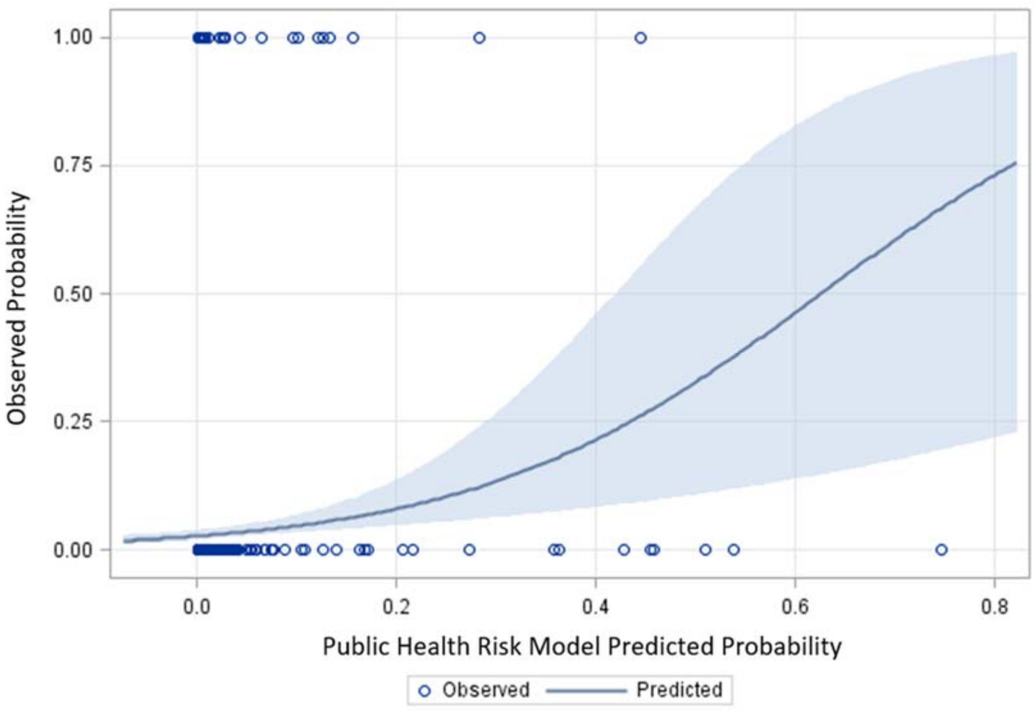

B. Histogram of Risk Model Probability by CRO Detection Status

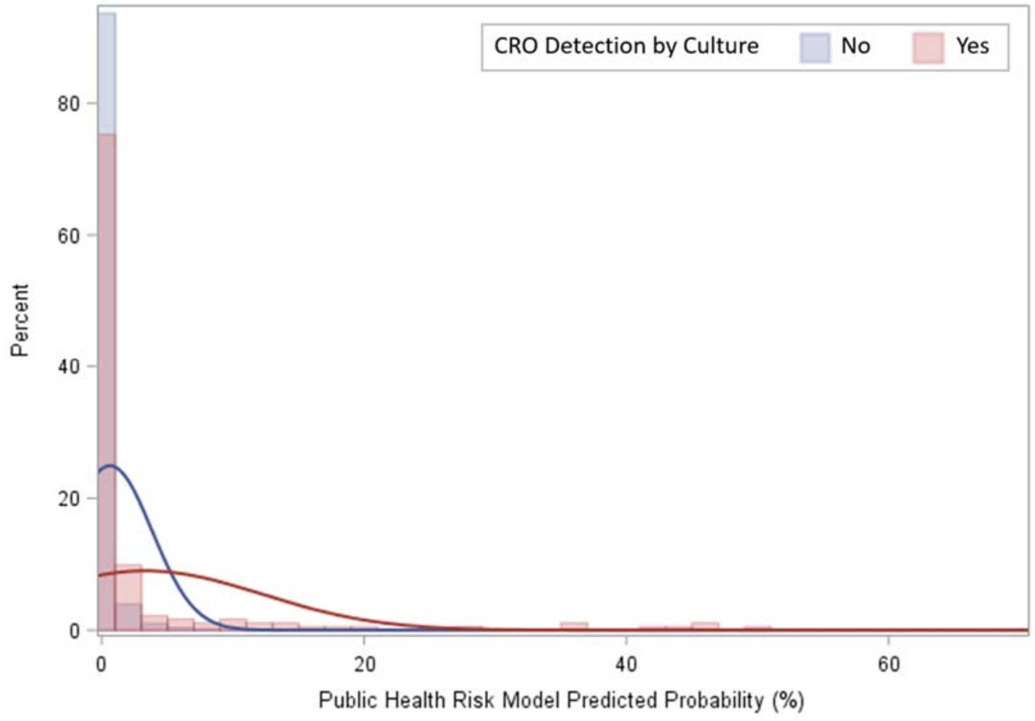

Supplement: Sansom et al. supplementary material [file S0899823X26103973sup001.pdf]
